# Natural polyphenol assisted delivery of single-strand oligonucleotides by cationic polymers

**DOI:** 10.1038/s41434-020-0151-y

**Published:** 2020-05-04

**Authors:** Wanwan Shen, Ruojun Wang, Qianqian Fan, Yiwen Li, Yiyun Cheng

**Affiliations:** 1 Shanghai Key Laboratory of Regulatory Biology, East China Normal University, Shanghai, 200241 China; 2 College of Polymer Science and Engineering, State Key Laboratory of Polymer Materials Engineering, Sichuan University, Chengdu, 610065 China; 3 South China Advanced Institute for Soft Matter Science and Technology, School of Molecular Science and Engineering, South China University of Technology, Guangzhou, 510640 China

**Keywords:** Gene delivery, Gene therapy

## Abstract

Single-strand oligonucleotides provide promising potential as new therapeutics towards various diseases. However, the efficient delivery of oligonucleotide therapeutics is still challenging due to their susceptibility to nuclease degradation and the lack of effective carriers for condensation. In this study, we reported the use of natural polyphenol to facilitate the condensation of single-strand oligonucleotides by cationic polymers. Green tea catechin complexed with single-strand oligonucleotides to form anionic nanoparticles, which were further coated by low molecular weight cationic polymers to increase their cell internalization. The resulting core-shell structured nanoparticles, so-called green nanoparticles (GNPs), showed improved cargo stability, and achieved high efficiency in the delivery of several types of single-strand oligonucleotides including antisense oligonucleotides, anti-miRNA, and DNAzyme. This study provides a facile strategy for the efficient delivery of single-strand oligonucleotides.

## Introduction

Oligonucleotide therapeutics including antisense oligonucleotides (ASOs), splice-switching oligonucleotides, steric blockers, aptamers, small Interfering RNA (siRNA), microRNA (miRNA), and other subtypes have shown enormous potential in the treatment of various diseases [1–3]. Since the first ASO was reported to inhibit the expression of sarcoma virus mRNA, oligonucleotide therapeutics have achieved great promise in clinical applications [4, 5]. Up to now, seven types of oligonucleotide drugs have been approved by FDA, namely fomivirsen (antisense nucleotide), pegaptanib (nucleic acid aptamer), mipomersen (antisense nucleotide), eteplirsen (antisense nucleotides), defibrotide (deoxyribonucleic acid derivatives), nusinersen (antisense nucleotides), and patisirna (siRNA). Note that four of them belong to single-strand oligonucleotides. Besides these drugs, there are still a large number of oligonucleotide drug candidates being widely evaluated in clinical studies [6, 7]. The common pharmacological challenges for single-strand oligonucleotides are their susceptibility to nuclease degradation, and massive dose requirement [1, 8]. During the past years, chemical modification and structural optimization on single-strand oligonucleotides have been proposed to overcome these limitations, i.e., the phosphate backbone of oligonucleotides was replaced by phosphorothioate, phosphodiamine morpholino, and peptide backbones [9–11]. These chemical strategies can alter the charge density and hydrophobicity of single-strand oligonucleotides, and thus improve their nuclease stability and base pairing efficiency.

However, chemically modified single-strand oligonucleotides usually possess relatively low membrane permeability, and limited stability during the long-term therapy [12]. To address those issues, the single-strand oligonucleotides were either conjugated with functional ligands, polymers, and nanoparticles [13, 14], or complexed with cationic polymers, liposomes, and nanomaterials [15] to increase their cell internalization, stability, and transfection efficiency. Cationic polymers with various chemical structures have widely used for intracellular delivery of biomolecules such as genes and proteins [16–21]. However, these materials have been usually puzzled by their unsatisfied correlations between transfection efficiency and cytotoxicity [22–24].

**Fig. 1:**
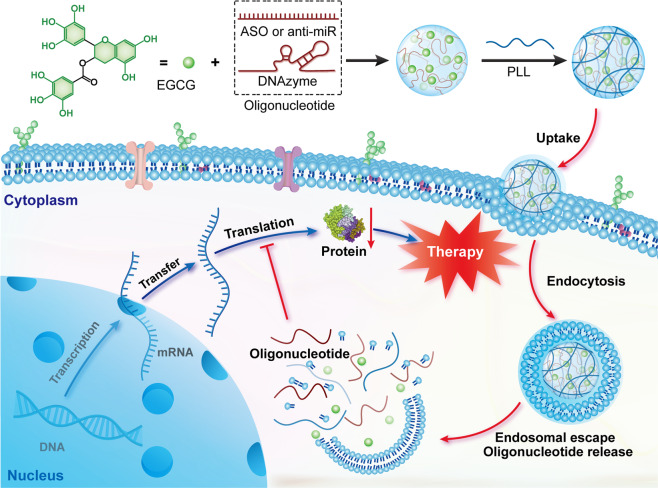
GNPs mediated gene delivery. Schematic illustration of GNPs for the delivery of single-strand oligonucleotides including ASO, anti-miRNA, and DNAzyme.

To break down the transfection efficiency–cytotoxicity correlation of polymers in gene delivery, we reported a facile and efficient siRNA delivery strategy using natural polyphenols and low molecular weight cationic polymers [25]. Natural polyphenols have strong binding affinity with various biomacromolecules such as proteins and nucleic acids via non-covalent interactions [26]. These molecules with potent antioxidant, antibacterial, and antitumor activities were widely used as synthons and function subunits to construct new functional materials for drug delivery [27–37]. One of the natural polyphenol (-)-epigallocatechin gallate (EGCG) was complexed with siRNA to form negatively charged nanoparticles, followed by surface coating on the nanoparticles with a shell of low molecular weight polymers such as ε-poly-L-lysine (PLL, from *Streptomyces albus*). EGCG protects the bound siRNA from nuclease degradation, and thus improves its stability during intracellular delivery, and the low molecular weight polymers on the particle surface enable efficient internalization but limited cytotoxicity. As a result, the prepared nanoparticles showed excellent gene-silencing efficiency both in vitro and in vivo. Considering that EGCG is the major component of green tea, and the nanoparticles are prepared by physical fabrication of several nontoxic compounds, this type of nanoparticles were termed green nanoparticles (GNPs). Since poor stability is the major challenge for single-strand oligonucleotides in gene therapy and most oligonucleotide drugs possess similar physicochemical properties, we hypothesized that this GNPs strategy may be also applicable for the delivery of single-strand oligonucleotides such as ASO, miRNAs, and DNAzymes (Fig. 1).

## Materials and methods

### Materials

EGCG, rhodamine B isothiocyanate (Rho), and 3-(4,5-dimethylthiazol-2-yl)-2,5-diphenyltetrazolium bromide (MTT) were obtained from Sigma-Aldrich (St. Louis, MO). PLL (4224 Da) was purchased from Macklin (Shanghai, China). Rabbit monoclonal antibodies against Bcl-2 (catalog number: ab182858) and β-actin (catalog number: ab16039) were purchased from Abcam, Inc. (Cambridge, UK). Trizol was obtained from NCM Biotech. (Suzhou, China) and Master-mix with SYBR-green kit was obtained from Takara, Inc. (Japan). Commercial transfection reagent Lipofectamine 2000 (LPF) was purchased from Thermo Fisher Scientific, Inc (Shanghai, China) and TransExcellent-siRNA (TE) was purchased from Cenji Biotech. (Shanghai, China). RNase and RNase Inhibitor were obtained from Yeasen Biotech. (Shanghai, China). GelRed was obtained from Beyotime Biotech. (Shanghai, China).

ASO, Ps-ASO, and siRNA specifically targeting firefly luciferase, glyceraldehyde-3-phosphate dehydrogenase (GAPDH), and prolyl hydroxylase-2 (PHD2), Bcl-2 DNAzyme, anti-microRNA-155 (anti-miR-155), scrambled ASO nonspecific to any gene (Sc-ASO), and ASO labeled with carboxyfluorescein at the 5′ end (ASO–FAM) were synthesized by Gene Pharma (Shanghai, China). The sequences of synthesized oligonucleotides were listed in Supplementary Table S1. All the chemicals were used as received without further purification.

### Characterization of EGCG/ASO complexes

Overall, 0.5 μg of ASO (40 μM, 1.88 μL) was mixed with 5 μg of EGCG (4.36 mM, 2.5 μL) in 100 μL of diethyl pyrocarbonate-treated water for 20 min to form EGCG/ASO complexes. Then the sample was diluted to a total volume of 1 mL by water. The mean hydrodynamic size and zeta potential of yielded nanoparticles were determined by dynamic light scattering (DLS, Zetasizer Nano ZS90, Malvern). The morphology of the formed complexes was observed by transmission electron microscopy (TEM, HT7700, HITACHI, Japan).

### Preparation and characterization of GNPs containing ASO

EGCG/ASO complex containing 0.5 μg ASO was prepared as described above, followed by the addition of 5 μg PLL (0.24 mM, 5 μL). The mixture was incubated in 100 μL of water for 30 min to form the GNPs. The samples were further diluted to 1 mL with water. The formed GNPs were characterized by DLS and TEM as described above.

### RNase degradation assay

GNPs containing 0.5 μg ASO were prepared as described above. The dose of ASO is 0.5 μg (40 μM, 1.88 μL), the weight ratio of EGCG (10.91 mM) to ASO is 10:1, and the weight ratio of PLL (1.18 mM) to EGCG is fixed at 1:1. The volume of mixture in each tube was replenished to 10 μL with water. The prepared GNPs were equilibrated for 30 min and the GNPs were treated with 10 μg/mL RNase (1 mg/mL, 0.1 μL) for 20 min, and then the activity of RNase was blocked by 0.1 μL RNase inhibitor (40 U/μL). Overall, 11 μL mixtures were further run on an agarose gel at 90 V (1.5% w/v gel, 10 min) after the addition of 1 μL (100 mg/mL) sodium heparin. The gel was stained by GelRed and visualized by a UV illuminator and the bands were photographed using an UVIpro Gel documentation system. Gray scale calculation was performed using Image J software. Commercial reagents LPF and TE were tested as controls, and the doses were both 2 μL.

### Cell culture and gene-silencing experiments

HeLa, HeLa-Luc (HeLa cells stably expressing firefly luciferase), and A549 cells were cultured in DMEM containing 10% fetal bovine serum (FBS), 100 U mL^−1^ penicillin, and 100 mg mL^−1^ streptomycin at 37 °C and 5% CO_2_.

The cells were seeded in 24-well plates at a density of 10^4^ cells per well and cultured for 24 h before gene-silencing experiments (50% confluence). Overall, 1.88 μL oligonucleotides (40 μM, 0.5 μg ASO-Luci for HeLa-Luc cells, 0.5 μg ASO-GAPDH and ASO-PHD2 for HeLa cells, 0.8 μg DNAzyme-Bcl-2 for HeLa cells, and 0.5 μg anti-miR-155 for A549 cells) were mixed with freshly prepared 2.5 μL EGCG (4.36 mM) for 20 min (the EGCG/oligonucleotide weight ratio of 10:1 for ASO and anti-miR-155, and 6.25:1 for DNAzyme-Bcl-2), followed by incubation with PLL (0.24 mM, PLL/EGCG weight ratio of 1:1) to yield GNPs, and further diluted with 100 μL FBS-free media (10 mM HEPES buffer was added to maintain the medium pH at 7.4). The GNPs solutions were incubated at the room temperature for 30 min, and further added with 150 μL culture media before added with cells. The cells were cultured with GNPs for 6 h. After that, 500 μL media containing 10% FBS were added into the wells and the gene-silencing experiments were continued for 18 h. The final concentration of oligonucleotides in the culture medium was 100 nM. Three repeats were conducted for each sample in three independent experiments.

The luciferase activity in HeLa-Luc cells was measured as previous study [25]. The expression levels of GAPDH, PHD2, and Bcl-2 mRNA in the treated cells were characterized by real-time reverse transcription quantitative PCR (RT-qPCR). Overall, 0.5 μg extracted RNA in the treated cells lysis was reverse-transcribed into cDNA and quantitative analyzed by qPCR (QuantStudio 3 Real-Time PCR Systems, Thermo Fisher Scientific) with the specific primers and SYBR-green kit. The related primers for each target gene were shown in Supplementary Table S2.

The Bcl-2 protein levels in the treated cells was analyzed by western blot. Generally, 50 μg total protein per lane were separated on 12% SDS-PAGE gels and transferred onto a PVDF membrane. The membrane was incubated with rabbit monoclonal antibodies against Bcl-2 overnight at 4 °C, and further incubated with IRDye 800 donkey anti-rabbit (LI-COR, USA) for 1 h. The protein bands on the gel were visualized using an Odyssey CLx infrared imaging system (LI-COR, USA). β-actin was used as the loading control.

Cell apoptosis in the treated cells were measured by an annexin V-FITC/propidium iodide (PI) apoptosis detection kit. The treated cells were stained by FITC-labeled Annexin V and PI for 15 min at the room temperature in the dark. The samples were quantitatively analyzed by flow cytometry (BD FACS Calibur, San Jose).

### Statistical analysis

Adequate sample size was determined according to the previous studies [23, 25] that performed analogous experiments. Data are represented as the derive average ± standard deviation throughout the manuscript. The variance was similar between the groups that are being statistically compared. Comparisons of data from tests and controls were analyzed for statistical significance by a one-sided Student’s *t* test using MS Excel. For all, *p* < 0.05 was considered statistically significant. *p < 0.05; **p < 0.01; ***p < 0.001. In this study, experiments were performed on at least three independent occasions, and no randomization and blinding were used. These tests were chosen since they best match the assumptions of the experiments.

## Results and discussion

**Fig. 2:**
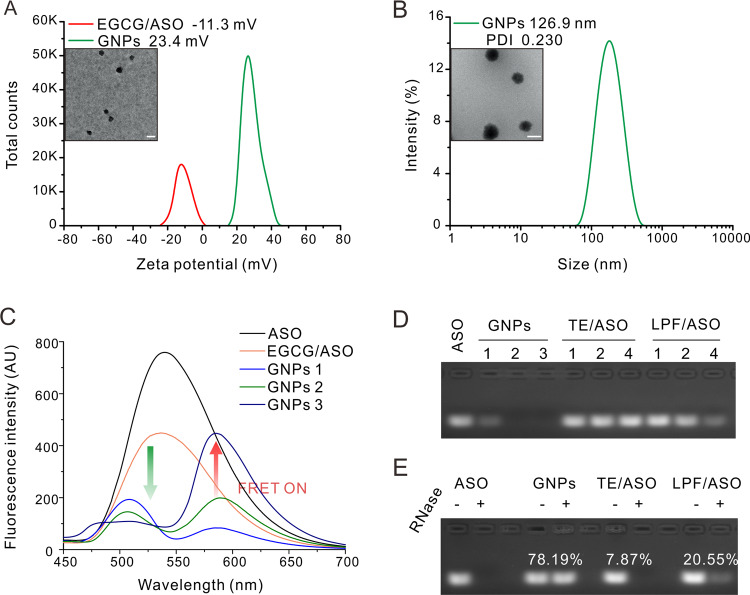
The characterizations of the prepared GNPs. **a** Zeta potential of EGCG/ASO complexes and GNPs. TEM image of EGCG/ASO complexes was insert in the picture. Scale bar is 100 nm. **b** DLS and TEM image of GNPs consisted of PLL, EGCG, and ASO. The dose of ASO was 0.5 μg (133 nM), EGCG to ASO weight ratio was 10:1, and PLL to EGCG weight ratio was 1:1. Scale bar is 100 nm. **c** Fluorescence spectra of ASO–FAM, EGCG/ASO–FAM, and GNPs consisted of ASO–FAM, EGCG, and PLL-Rho. The dose of ASO–FAM in each well was 1.0 μg (267 nM), the weight ratio of EGCG to ASO–FAM was 5:1, 10:1, 20:1 for GNPs 1, GNPs 2, and GNPs 3, respectively. PLL to EGCG weight ratio was 1:1 and EGCG to ASO–FAM weight ratio was 20:1 for the EGCG/ASO complex. **d** Gel electrophoresis of GNPs. The dose of ASO was 0.5 μg, and the weight ratio of EGCG to ASO was 5:1, 10:1, 20:1 for GNPs 1, GNPs 2, and GNPs 3, respectively. PLL to EGCG weight ratio was 1:1. TE/ASO and LPF/ASO complexes were tested as controls, and the doses of TE and LPF were 1, 2, and 4 μL. **e** Gel electrophoresis of RNase-treated GNPs. The numbers on the band represent the percent of retained ASO, which were calculated by ImageJ software. The material doses in GNPs were equal to those in GNPs 2 in (**d**). The doses of TE and LPF were 2 μL, and the concentration of RNase was 10 μg/mL.

We first investigated the formation of EGCG/single-strand oligonucleotide ASO complexes using DLS and TEM. It was found that EGCG and ASO formed negatively charged nanoparticles in aqueous solution (−11.3 mV, Fig. 2a). After coating with PLL, nanoparticles with an average hydrodynamic size of 127 nm were observed (Fig. 2b), and the zeta potential of particles increased from −11.3 to 23.4 mV (Fig. 2a), suggesting the successful coating of PLL on the EGCG/ASO complexes. We further investigated the complexation process by fluorescence resonance energy transfer (FRET) experiment. As shown in Fig. 2c, the fluorescence of carboxyfluorescein labeled ASO (ASO–FAM) was significantly decreasing after the addition of EGCG due to the formation of ASO–FAM complexes. After coating with rhodamine-labeled PLL (PLL-Rho), the fluorescence intensity of ASO–FAM was further decreasing, while that of PLL-Rho was increasing (Fig. 2c). The FRET signals increased with increasing EGCG/PLL to ASO weight ratios, and this result confirmed the formation of PLL-coated EGCG/ASO nanoparticles (GNPs: the PLL/EGCG/ASO weight ratio is 5:5:1, 10:10:1 and 20:20:1, respectively for GNPs 1–3) in the solutions. Note that the distinct fluorescence spectra of GNPs 1–3 were observed, which might be due to their different particle size and aggregated structures. We also investigated the ASO-binding capability of GNPs by gel electrophoresis. Two commercial gene transfection reagents TE (a polymer-based reagent) and LPF (a lipid-based reagent) were used as the controls. The ASO was efficiently bound in the nanoparticles when the EGCG to ASO weight ratio was above 10:1 (Fig. 2d), while TE and LPF failed to efficiently bind with ASO under the electrophoresis condition. We further investigated the protective effects of GNPs on ASO via RNase degradation experiment. The remained ASO in the GNPs was 78.19% after RNase (10 μg/mL) treatment for 20 min, while those of TE and LPF were 7.87% and 20.55%, respectively (Fig. 2e), which suggested that GNPs can efficiently protect ASO from degradation by RNase and increase the tolerance of ASO to nuclease. The increased stability of ASO in GNPs might be due to the core-shell structure of GNPs, in which the oligonucleotides located in the interior of formed nanoparticles [25]. Since GNPs 2 with the PLL/EGCG/ASO weight ratio of 10:10:1 showed effective ASO binding and complex stability, this formulation was further used in subsequent gene transfection experiments.

**Fig. 3:**
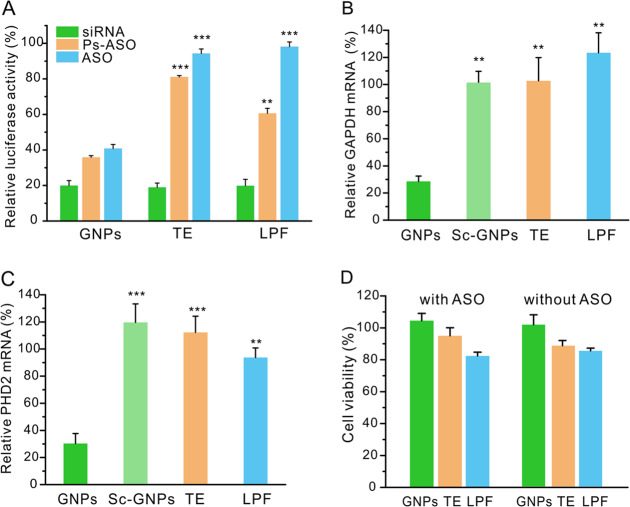
Efficiency of GNPs in the delivery of oligonucleotides. **a** Efficiency of GNPs in the delivery of siRNA, ASO, and Ps-ASO into Hela-Luc cells for 24 h. The concentration of oligonucleotides in each well was fixed at 100 nM. The dose of EGCG in each well was 5 μg, and the weight ratio of PLL to EGCG was 1:1. GAPDH (**b**) and PHD2 (**c**) knockdown efficiency of GNPs in HeLa cells. GNPs were consisted of ASO, EGCG, and PLL. The concentration of ASO was 100 nM (1.0 μg), the weight ratio of EGCG to ASO was 10:1, and the weight ratio of PLL to EGCG was 1:1. Data are representative of three independent experiments performed with three technical duplicates in (**a**–**c**). **d** Cell viability of HeLa cells treated with GNPs and EGCG/PLL for 24 h. The concentrations of the materials were equal to those in (**b**, **c**). TE and LPF were tested as controls. The doses of TE and LPF in each well were both 2.0 μL. Data are representative of three independent experiments performed with five technical duplicates. **p < 0.01, ***p < 0.001 analyzed by Student’s *t* test.

We then tested the efficiency of GNPs in the delivery of ASO into HeLa-Luc cells to knockdown the firefly luciferase gene. Three type of oligonucleotides including non-modified ASO, phosphorothioate backbone ASO (Ps-ASO), and double-strand siRNA targeting luciferase were tested as cargo molecules. The antisense strand of these three oligonucleotides is identical to each other and the concentration of oligonucleotides was fixed at 100 nM. As shown in Fig. 3a, GNPs, TE, and LPF exhibited high gene-silencing efficiency in the delivery of siRNA. However, the gene knockdown efficiencies by TE was decreased to 19% and 6% when Ps-ASO and ASO were used as the cargo oligonucleotides, and the efficiencies of LPF was decreased to 39% and 2%, respectively. On the contrary, GNPs still maintained relatively high efficiency in the delivery of Ps-ASO (64%) and unmodified ASO (59%). The delivery efficiency of these oligonucleotides by the materials was in good agreement with their stability. The high efficiency of GNPs in the delivery of single-strand ASO and Ps-ASO should be attributed to the excellent stability of single-strand oligonucleotides in GNPs as well as the efficient endocytosis and intracellular release [25]. We subsequently tested the efficiency of GNPs in the delivery of ASOs targeting different genes on HeLa cells. As shown in Fig. 3b, c, GNPs significantly knocked down the expressions of GAPDH and PHD2 in the cells when delivering single-strand ASO targeting these genes, while TE, LPF, and GNPs containing scrambled ASO (Sc-GNPs) exhibited low gene silencing on the cells. In addition, the GNPs with or without ASO did not cause obvious cytotoxicity at the transfection concentrations (Fig. 3d).

**Fig. 4:**
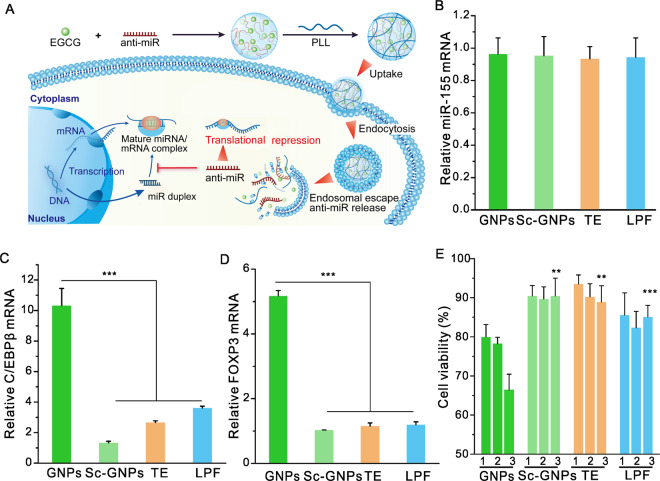
Efficiency of GNPs in the delivery of anti-miRNA into A549 cells. **a** Schematic illustration of anti-miRNA delivery by GNPs. The expression levels of miR-155 (**b**), C/EBPβ (**c**) and FOXP3 (**d**) in GNPs-treated A549 cells for 24 h. The dose of anti-miR-155 was 0.5 μg in each well (100 nM), the weight ratio of EGCG to anti-miR-155 was 10:1, and the weight ratio of PLL to EGCG was 1:1. ***p < 0.001 analyzed by Student’s *t* test. Results represent three independent biological PCR reactions performed in triplicate. in (**b**–**d**). **e** Viability of GNPs-treated A549 cells for 24 h. The doses of anti-miR-155 in 1, 2, 3 were 0.5 μg in each well (100 nM), 1.0 μg (200 nM), and 1.5 μg (300 nM), respectively. The dose of EGCG was 5.0 μg, the weight ratio of PLL to EGCG was 1:1, and the doses of TE and LPF were 2.0 μL. Data are representative of three independent experiments performed with five technical duplicates. **p < 0.01, ***p < 0.001 between GNPs 3 and other control groups analyzed by Student’s *t* test.

miRNA usually inhibits the translation of target mRNA by complementary pairing, and its function can be inhibited by anti-miRNA (anti-miR), a type of steric blocker ASOs [38–40] (Fig. 4a). MiR-155, a typical multifunctional miRNA, is related to tumorigenesis and metastasis in a variety of cancer cells and has been recognized as a novel tumor biomarker for cancer therapy [41, 42]. Anti-miRNA-155 can be used to inhibit the function of oncogenic miRNA-155. The pre-complementary pairing of anti-miRNA with miRNA hinders the recognition of oncogene miRNA-155 with its target mRNA such as transcription factor CCAAT enhancer binding protein β (C/EBPβ) and forkhead transcription factor FOXP3 to prevent it from functioning properly [43]. Herein, GNPs were further used to deliver anti-miR-155 to inhibit the function of miR-155. The mRNA expression level of miRNA-155 was scarcely changed after treatment (Fig. 4b), but the levels of target gene C/EBPβ and FOXP3 in the cells were increased by tenfold and fivefold, respectively (Fig. 4c, d), which were again significantly higher than those by control materials (TE and LPF). Sc-GNPs containing nonsense anti-miRNA did not cause significant changes in the expressions corresponding target genes. The results suggested that anti-miR-155 was successfully delivered into cells and specifically bound with miR-155 to inhibit its function. As a result, the viability of A549 cells was significantly reduced (Fig. 4e). All the evidences above demonstrated that GNPs can efficiently deliver anti-miRNA into cells to regulate the expressions of downstream genes.

**Fig. 5:**
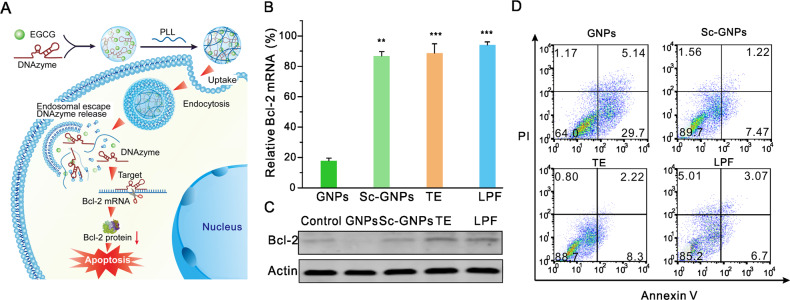
Efficiency of GNPs in the delivery of DNAzyme into HeLa cells. **a** Schematic illustration of Bcl-2 DNAzyme delivery by GNPs. **b** Bcl-2 mRNA expression in GNPs-treated HeLa cells for 24 h. Results represent three independent biological PCR reactions performed in triplicate. **c** Representative Bcl-2 protein expressions in the treated cells. **d** Flow cytometry analysis of treated HeLa cells stained by annexin V and propidium iodide. The concentration of DNAzyme in each well fixed at 100 nM (0.8 μg), the dose of EGCG and PLL in each well was both 5.0 μg. The doses of TE and LPF were 2.0 μL. **p < 0.01, ***p < 0.001 analyzed by Student’s *t* test.

In comparison with ASOs, DNAzyme has lower off-target effects due to its special internal structure, which consists of both binding sites and catalytic sites within the molecule. Numerous clinical results have pointed out that DNAzyme is well-tolerated in humans [44–46]. Bcl-2 is an oncogene and numerous researches have been focused on the downregulation of Bcl-2 by oligonucleotides to induce apoptosis in cancer cells [47–49]. We finally tested the efficiency of GNPs in the delivery of DNAzyme targeting Bcl-2 on HeLa cells (Fig. 5a). As shown in Fig. 5b, GNPs containing the DNAzyme significantly reduced the expression of Bcl-2 mRNA, with an efficiency higher than 80%. However, GNPs containing nonsense DNAzyme failed to downregulate the target gene, indicating the successful delivery of Bcl-2 DNAzyme into the cells. Furthermore, the GNPs containing Bcl-2 DNAzyme successfully inhibited Bcl-2 protein levels (Fig. 5c) and caused significantly increased apoptosis in the cells (Fig. 5d).

## Conclusions

Inspired by the high efficiency of GNPs in siRNA delivery, we investigated the behaviors of GNPs in the delivery of several single-strand oligonucleotides including ASO, anti-microRNA, and DNAzyme. The results revealed that GNPs can efficiently protect the single-strand nucleic acids from enzymatic degradation, and successfully delivered these oligonucleotides into cells to exert their biofunctions. This study further confirmed that GNPs could be developed as a facile, robust, and efficient strategy in the delivery of oligonucleotide therapeutics. Our future work will test the efficiency of these GNPs in the delivery of single-strand oligonucleotides in vivo and evaluate their potentials in clinical translation.

## Supplementary information

supporting information

## Data Availability

All relevant data generated in this study are included in the published article and supplementary information.
