# Supplementary material for: Natural polyphenol assisted delivery of single-strand oligonucleotides by cationic polymers
Source: Gene Ther. 2020 May 4;27(7):383–91. doi: 10.1038/s41434-020-0151-y (PMC7445782; doi:10.1038/s41434-020-0151-y)
Supplement: Supplementary file 1 — supporting information [file 41434_2020_151_MOESM1_ESM.docx]

**Supporting Information**

**Natural Polyphenol Assisted Delivery of Single-strand Oligonucleotides by Cationic Polymers**

Wanwan Shen^1^, Ruojun Wang^1^, Qianqian Fan^1^, Yiwen Li^2*^, Yiyun Cheng^1,3^*

^1^Shanghai Key Laboratory of Regulatory Biology, East China Normal University, Shanghai 200241, China.

^2^College of Polymer Science and Engineering, State Key Laboratory of Polymer Materials Engineering, Sichuan University, Chengdu, 610065, China.

^3^South China Advanced Institute for Soft Matter Science and Technology, School of Molecular Science and Engineering, South China University of Technology, Guangzhou 510640, China.

*Correspondence may be addressed to [yycheng@mail.ustc.edu.cn](mailto:yycheng@mail.ustc.edu.cn), ywli@scu.edu.cn.

**Experimental section**

**Fluorescence measurement**

1.0 μg ASO labelled with carboxyfluorescein at the 5' end (ASO-FAM, 40 μM, 3.76 μL) was complexed with (-)-epigallocatechin gallate (EGCG, 4.36 mM) at different weight ratios (EGCG/ASO-FAM = 5:1, 10:1, 20:1 for GNPs 1, GNPs 2 and GNPs 3, respectively). The complex solutions were further added with rhodamine-labeled ε-poly-L-lysine (PLL-Rho, 0.24 mM, PLL/EGCG weight ratio of 1:1). The formed GNPs in 1.0 mL diethyl pyrocarbonate (DEPC)-treated water and incubated for 30 min in the dark before measurement by a fluorescence spectrometer (F-4500, HITACHI, Japan). The excitation wavelength was 420 nm and the fluorescence spectroscopy were recorded at 450-700 nm. The fluorescence spectra of ASO-FAM, and ASO/EGCG (EGCG/ASO-FAM = 20:1) were tested as controls.

**Agarose gel electrophoresis**

GNPs containing ASO were prepared as described above. The dose of ASO is 0.5 μg (40 μM, 1.88 μL), the weight ratios of EGCG (10.91 mM) to ASO are 5:1, 10:1, 20:1, respectively for GNPs 1-3, and the weight ratio of PLL (1.18 mM) to EGCG is fixed at 1:1. The volume of mixture in each tube was replenished to 10 μL with DEPC water. The total 10 μL solution was equilibrated for 30 min and run on a 1.5% (w/v) agarose gel under 90 V for 10 min. The gel was stained by GelRed, and observed by an UVIpro Gel documentation system (Tanon-2500, China).

**Cytotoxicity analysis**

Viability of treated cancer cells was measured by a well-established MTT assay. Cells cultured in 96-well plates at a density of 10000 cells per well were incubated with GNPs for 24 h, 0.5 μg ASO (40 μM) was mixed with freshly prepared 2.5 μL EGCG (4.36 mM) for 20 min, followed by incubation with PLL (0.24 mM, PLL/EGCG weight ratio of 1:1) to yield GNPs, and further diluted with 100 μL cell culture media (10 mM HEPES buffer was added to maintain the medium pH at 7.4). The cell viability was measured according to the manufacturer’s protocol. Commercial reagents Lipofectamine 2000 (LPF) and TransExcellent-siRNA (TE) were tested as controls, and the doses were both 2 μL, and the materials only (without ASO) were test in parallel experiment. Five repeats were conducted for each sample in three independent experiments.

**Statistical analysis**

Adequate sample size was determined according to the previous studies 23,25 that performed analogous experiments. Data are represented as the derive average ± standard deviation (S.D.) throughout the manuscript. The variance was similar between the groups that are being statistically compared. Comparisons of data from tests and controls were analyzed for statistical significance by a one-sided Student’s t-test using MS Excel. For all, p < 0.05 was considered statistically significant. *p < 0.05; **p < 0.01; ***p < 0.001. In this study, experiments were performed on at least three independent occasions, and no randomization and blinding were used. These tests were chosen since they best match the assumptions of the experiments.

**Table S1**. Sequences of oligonucleotides in this study.

| Gene | Sense (5’-3’) | Antisense (5’-3’) |
| --- | --- | --- |
| ASO-GAPDH |  | CUUGAGGCUGUUGUCAUACdTdT |
| ASO-PHD2 |  | AUUUGGGUUAUCAACGUGAdTdT |
| ASO-Luci |  | GCGAAGAAGGAGAAUAGGGdTdT |
| siRNA-Luci | CCCUAUUCUCCUUCUUCGCdTdT | GCGAAGAAGGAGAAUAGGGdTdT |
| Bcl-2 DNAzyme |  | CACAGCCAAGGCTAGCATCAACGA  GTGCCATGT |
| Anti-miR-155 |  | a*c*ccuaucacgauuagcauu*a*a |

*Represents a phosphorothioate backbone, lowercase letter represents 2’-O-Me modification on the base. The sequence for Ps-ASO and ASO-Luci is the same except that the phosphate backbone on Ps-ASO is replaced by phosphorothioate.

**Table S2** Sequences of primers in this study.

| Gene | Forward (5’-3’) | Reward (5’-3’) |
| --- | --- | --- |
| GAPDH | GTCAGTGGTGGACCTGACCT | ACCTGGTGCTCAGTGTAGCC |
| MMP-9 | AAGATGCTGCTGTTCAGCGGG | GTCCTCAGGGCACTGCAGGAT |
| PHD2 | AGCTGGTCAGCCAGAAGAGT | GCCCTCGATCCAGGTGATCT |
| Bcl-2 | GGACACGGACAGGATTGACA | GACATCTAAGGGCATCACAG |
| miR-155 | CTCAGACTCGGTTAATGCTAATC  GTGATAGG | GCTGTGGCAGTGGAAGCGTGAT  TTATT |
| C/EBPβ | AGAAGACCGTGGACAAGCACAG | TTGAACAAGTTCCGCAGGGTGG |
| FOXP3 | AATGGCACTGACCAAGGCTTC | TGTGGAGGAACTCTGGGAATGTG |
| 18s | GACATCTAAGGGCATCACAG | GGACACGGACAGGATTGACA |
